# Hodgkin’s Disease

**Published:** 1914-06

**Authors:** 


**Hodgkin's Disease.** Bunting and Yates, Archives of Int. Med., 1913, No. 12, have isolated, in three cases, a pure culture of a pleomorphic, diphtheroid organism. Previous descriptions of similar organisms have been made by Negri and Mieremet, also by Fraenkel and Munch.

